# Relationship between bladder cancer and total fluid intake: a meta-analysis of epidemiological evidence

**DOI:** 10.1186/1477-7819-12-223

**Published:** 2014-07-17

**Authors:** Yunjin Bai, Haichao Yuan, Jinhong Li, Yin Tang, Chunxiao Pu, Ping Han

**Affiliations:** 1 Department of Urology, West China Hospital, Sichuan University, Guoxue Xiang#37, Chengdu, Sichuan 610041, China

**Keywords:** Bladder neoplasms, Fluid consumption, Etiology, Meta-analysis

## Abstract

**Objectives:**

Epidemiological findings regarding the association between total fluid intake and bladder cancer risk have yielded varying results. Our objective is to examine the possible associations between total fluid intake and bladder cancer risk.

**Methods:**

Databases searched include the EMBASE and PUBMED, from inception to February 2014, with no limits on study language. We also reviewed the reference lists of identified studies. Stratified analyses were performed. A random-effect model was used to summarize the estimates of odds ratio (OR) with 95% confidence intervals (CI).

**Results:**

Overall,17 case-control and four cohort studies were included. The overall OR of bladder cancer for the highest versus the lowest fluid intake was 1.06 (95% CI: 0.88-1.27). In the subgroup analyses, the overall ORs for coffee, green, and black tea intake were 1.17 (95% CI: 1.03-1.33), 0.76 (95% CI: 0.66-0.95), and 0.80 (95% CI: 0.65-0.97), respectively. A significantly decreased risk was observed in Asian people (OR 0.27; 95% CI: 0.10-0.72). Among smokers, a suggestive inverse association was observed between total fluid intake and overall bladder cancer risk (OR 0.80; 95% CI: 0.62-1.02).

**Conclusions:**

Although this meta-analysis suggested that greater consumption of fluid may have a protective effect on bladder cancer in Asian people, there was no convincing evidence on this association because of the limitations of the individual trials.

## Review

### Introduction

Bladder cancer is one of the most common malignant tumors in the United States, with an estimated 74,690 new cases and 15,580 deaths in 2014 [1]. Approximately 30% of cases present as an invasive muscle bladder cancer, which has a poorer prognosis [2]. To date, feasible measures for the prevention of bladder cancer remain limited. A better understanding of the etiology of bladder cancer may lead to marked reductions in both the incidence and mortality.

The cause of bladder cancer is not well-known, but multiple risk factors have been identified, including tobacco use, occupational exposure to chemicals found in the chemical and rubber industries, schistosomal chronic infection, and high concentrations of arsenic in the drinking water [3]. In recent years certain medications have been investigated in several studies. Results suggested that pioglitazone treatment was associated with an increased risk of bladder cancer in patients with type 2 diabetes, and that exposure to herbs containing aristolochic acids has also been associated with bladder cancer [4,5]. As for other cancers, dietary aspects have been responsible for the occurrence and development of bladder cancer. Some research has shown that processed meat consumption may increase the risk [6], and the intake of fruits and vegetables may decrease the risk [7].

Fluid intake is also commonly evaluated because of its impact on voiding, but the association with bladder cancer remains controversial. To date, studies on the relationship between fluid intake and bladder cancer risk have yielded inconsistent results. On the one hand, the amount of fluid consumption may reduce exposure of bladder epithelium to carcinogens by diluting urine and increasing the urination frequency. On the other hand, the type of fluid is related to the risk if it is contaminated with carcinogens, such as chlorination byproducts or arsenic [8]. In 2006, a pooled analysis suggested that high fluid consumption may increase the risk of bladder cancer [9], whereas recent findings from case-control study and a randomized controlled trial did not support such an association [10,11]. Therefore, a synthesis of the current evidence is needed.

To this end, we conducted a meta-analysis of all published case-control and cohort studies to assess the relationship between total fluid consumption and bladder cancer risk.

## Methods

According to the Meta-Analysis of Observational Studies in Epidemiology guidelines for study reporting [12], a prospective protocol of objectives, literature search strategies, eligibility criteria, and methods of statistical analysis was prepared.

### Literature search

In accordance with a pre-specified study protocol, a comprehensive electronic database search of PubMed and EMBASE was performed to identify articles published up to February 2014. Search terms included ‘fluid or water or diet or dietary’ and ‘bladder cancer or urothelial cancer or transitional or bladder neoplasm or bladder carcinoma’. No language limitations were imposed. We evaluated potentially relevant publications by examining their titles and abstracts and all of the studies matching the eligibility criteria were included. Moreover, the bibliographies of all studies included were manually searched to identify additional studies.

### Inclusion criteria

For a study to be included, in this meta-analysis, it was necessary for it to meet all of the following criteria: (1) case-control or cohort study assessing the relationship between total fluid intake and bladder cancer risk, (2) exact data in both case and control groups (participants for cohort studies) should be determined and (3) results including adjusted effect estimates with their 95% CIs or sufficient information allowing us to calculate them. Studies with overlapping or insufficient data were excluded. Letters, abstracts, editorials, animal trails, and literature reviews were excluded from this meta-analysis. Throughout the process, any questions or discrepancies were resolved by the consensus of all authors.

### Data extraction

According to the pre-specified protocol, all data were extracted independently by two authors. We extracted the following data from each eligible study by using a standardized data collection form: the first author’s name, the year of publication, the country where the study was conducted, sex, sample size, the adjustment for potential confounders, and effect estimates comparing the highest level of total fluid intake with the lowest. Given that bladder cancer is a rare disease, the relative ratio (RR) was assumed to be the same as the odds ratio (OR). Consequently, we report all results as ORs. The overall fluid definition as ‘fluid intake’ or ‘total fluid’ included fluid defined in the publications. To avoid residual confounding by smoking and to assess effects by smoking strata, we also extracted ORs for non-smokers.

### Methodological quality assessment

The Newcastle-Ottawa Scale (NOS) [13] was used to assess the methodological quality of cohort and case-control studies on three broad perspectives: selection, comparability, and exposure or outcome. Two authors read each study and scored them independently. Disagreement between the two authors was settled by discussion with the third author and resolved by consensus.

### Statistical analysis

The ORs were abstracted from each included study and then transformed to their natural logs. The log of the ORs was weighted by the inverse of their variances to obtain a pooled OR with 95% CI. Statistical heterogeneity among studies was measured using the Q-test [14] and calculating the I^2^ score [15]. For the Q-test, heterogeneity was considered present for *P*<0.10. The I^2^ exceeding 50% is considered to indicate the presence of heterogeneity. In cases of a lack of heterogeneity, the Mantel-Haenszel fixed-effect model was used to provide summary estimations of the total fluid intake associated with bladder cancer risk, otherwise, the DerSimonian and Laird random-effect model was used for the meta-analysis [14,16].

Publication bias was assessed through the visual inspection of funnel plots and with tests of Begg rank correlation [17] and Egger regression asymmetry [18]. *P*<0.05 was considered to be representative of a significant statistical publication bias. To explore the potential heterogeneity among studies, subgroup analyses were conducted according to gender (male and female), study design (cohort and case-control studies), geographical region (Europe and Asia), smoking status (non-smoker and smoker), publication year (before2000 and after 2000), type of beverage (water, coffee, tea and alcoholic beverages), and number of beverages. Statistical significance was set at *P*<0.05. STATA version 12.0software was used for the statistical analyses (StataCorp, College Station, Texas, United States).

## Results

### Search result and characteristics of included studies

**Figure 1:**
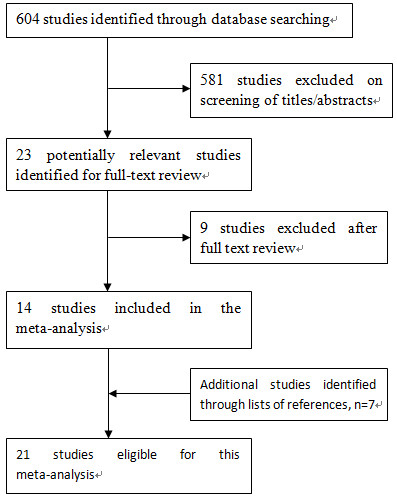
Flow diagram of studies identified, included, and excluded.

Using the predefined search strategies, we ultimately included 21 articles [9,10,19-37] that investigated the association of total fluid intake with bladder cancer risk, including 17 case-control [9,10,19-33] and four cohort studies [34-37]. The flow diagram (Figure 1) showed the detailed literature search steps. Among the 17 case-control studies, 9 studies [10,21,23,25,27-29,31,33] reported 2 separate outcomes (male and female). Of the four cohort studies, one study [36] reported two separate outcomes (male and female). Six studies [10,21,29,34-36] were eligible for pooling estimates of total fluid intake among non-smokers (three cohort studies). Baseline characteristics of the eligible studies are presented in Tables 1 and 2. Of these studies, 17 [10,19,23-36] were conducted in Europe and North America (9 in the United States, 8 in other countries) and 4 [10,20-22] in Asia.

**Table 1:** Study characteristics of published case–control studies of total fluid intake and bladder cancer risk

**References, year of publication**	**Country**	**Sex**	**Number of cases/ controls**	**Total fluid consumption**	**Study quality**	**Effect estimates**	**Adjustment factors**	**Observations**
Wang *et al.* 2013 [19]	USA	F/M	1,007/1,299	≥2789 vs. <1696 ml/day	8	1.41 (1.10-1.81)	Age, sex, ethnicity, energy intake, and smoking.	Coffee, tea, water, alcoholic beverage, soft drink
Ahmad *et al*. 2012 [20]	Pakistan	F/M	55/99	≥10 vs. <10 glasses/day	6	0.116 (0.04-0.33)	NA	Coffee, tea, alcohol
Zhang *et al*. 2010 [10]	China	F/M	608/607	>1500 vs. ≤750 ml/day	8	0.89 (0.65-1.22)	Age, sex, smoking status, BMI, bladder infections, high-risk occupation, alcohol drinking, urinate status	Coffee, tea, water, beverage , alcohol
Hemelt *et al*. 2010 [21]	China	F/M	432/392	The highest vs. lowest quintile	7	0.65 (0.43-0.98)	Age, sex, smoking status, smoking frequency, smoking duration	Black tea, green tea, fruit juice, milk, soft drinks and water, beer, wine and liquor/spirits
						F: 2.19 (0.89-5.38)		
						M:0.58 (0.37-0.92)		
Ahmad and Pervaiz 2010 [22]	Pakistan	F/M	50/100	≥10 vs. <10 glasses/day	6	0.025 (0.005-0.115)	NA	Coffee, tea, alcohol
Jiang *et al*. 2008 [23]	USA	F/M	1,586/1,586	The highest vs. lowest quartile	6	0.98 (0.77-1.26)	Education, NSAIDs, intake of carotenoids, hairdresser/barber, cigarette smoking status, duration and intensity of smoking	Water, coffee, tea, alcohol, milk, juice, hot chocolate, and soda
						F:1.19 (0.67-2.09)		
						M:0.93 (0.71-1.24)		
Michaud *et al*. 2007 [24]	Spain	F/M	397/664	The highest vs. lowest quintile	7	0.62 (0.40-0.95)	Age, sex, region, cigarette smoking, high-risk occupation, nighttime urination frequency, THM levels, non-tap fluid for water intake	Coffee, beer, wine, liquor, champagne, soda, juices, tea, milk, and water
Villanueva *et al*. 2006 [9]	Europe, America	F/M	2729/5150	>3.5 vs. ≤2 l/day	8	F:1.06 (0.77-1.46)	age, gender, study, smoking status, occupation, and education	Tap water, coffee, non-coffee-tap water, non-tap water
						M:1.33 (1.12-1.58)		
Geoffroy-Perez and Cordier 2001 [25]	France	F/M	765/765	F: >12800 vs. ≤7300 ml/week M: >16800 vs. ≤8300 ml/week	6	F: 0.96 (0.42-2.22)	Age, center, and place of residence, and smoking	Tap water, coffee, tea, bottled alcohol
			F: 106/106					
			M: 602/615			M: 1.07 (0.72-1.59)		

Bianchi *et al*. 2000 [26]	USA	F/M	1,452/2,434	≥2.6 vs. <2.6 l/day	7	1.32 (1.16-1.51)	Age, education, smoking, pack-years of smoking, family history of bladder cancer, high risk occupation, total beverage consumption, years of chlorinated surface water, vegetable, coffee consumption	Water, coffee, tea, fruit juices/drinks, soups, milk, soft drinks, and alcoholic beverages
Pohlabeln *et al*. 1999 [27]	Germany	F/M	F:61/61	F:>2 l/day vs. <1 l/day M:>3 l/day vs. <1 l/day	6	F:0.34 (0.11-0.99)	Smoking	Coffee, tea, water, wine, bottles of beer, and other beverages
			M:239/239					
						M:1.52 (0.64-3.59)		
Bruemmer *et al*. 1997 [28]	USA	F/M	262/405	>12 vs. ≤7 cups/week	6	F: 4.7 (1.4-15.8)	Age, county, and smoking	Water, coffee, decaffeinated coffee, tea, diet soft drinks, regular soft drinks, wine, beer, and liquor
						M: 1.0 (0.5-1.7)		
Wilkens* et al*. 1996 [29]	USA	F/M	F: 66/132	The highest vs. lowest quartile	7	F: 0.3 (0.1-0.8)	Age, smoking status, pack-years, high-risk occupation, dark green vegetables in men, total vitamin C consumption in women	Coffee, black and green tea, soda, beer, spirits, wine, fruit juice, cocoa, water, and milk
			M: 195/390			M: 1.4 (0.8-2.6)		
Vena *et al*. 1993[30]	USA	F/M	351/855	The highest vs. lowest quartile	8	3.74 (2.55-5.47)	Age, education, cigarette smoking, other liquids, sodium, carotene, and calories	Alcoholic beverages, bottled beverages, soda, milk, coffee, tea, all juices, and glasses of tap water
Kunze*et al*. 1992 [31]	Germany	F/M	F:75/71	F:1.1-2.0 vs.2.1-3.0 l/day	6	F:0.9 (0.3-2.5)	Smoking	Coffee, tea, beer, high-proof spirits, wine, all nonalcoholic, alcoholic beverages
			M:416/360	M:1.1-2.0vs. >3.1 l/day		M:4.9 (2.0-12.3)		
Slattery *et al*. 1988 [32]	USA	F/M	419/889	>653 vs. ≤289 oz./week	6	1.36 (0.89-2.07)	Age, sex, diabetes, bladder infections, and cigarette smoking	Coffee, tea, soft drinks, water, milk, cocoa, chocolate milk, fruit and vegetable juice, other non-alcoholic beverages, alcoholic beverages
Jensen *et al*. 1986 [33]	Denmark	F/M	371/771	F: 3-3.99 vs. 0-0.99 l/day	7	F:1.8 (0.4-1.4)	Smoking	Coffee, tea, beer, and soft drinks as well as other beverages
				M:≥4.00vs. 0-0.99 l/day		M:3.3 (1.4-7.4)		

F: female; M: male; NA: not available; NSAIDS: non-steroidal anti-inflammatory drugs; THM: trihalomethane.

**Table 2:** Study characteristics of published cohort studies of total fluid intake and bladder cancer risk

**References, year of publication**	**Country**	**Sex**	**Number of participants/cases**	**Total fluid consumption**	**Study quality**	**Effect estimates**	**Adjustment factors**	**Observations**
Zhou *et al*. 2014 [34]	USA	F	160,041/427	The highest vs. lowest quartile	7	0.83 (0.61-1.12)	Age in years, pack-years of smoking (5 categories), current smoking status (yes *vs* no), consumption of bacon (3 categories), energy intake (in quartiles), and intake of fruit and vegetables (in quartiles)	Water and specific beverages
Zhou *et al.* 2012 [35]	USA	F/M	924,221/823	>2,531 vs. < 1,290 ml/day	7	1.02 (0.79-1.32)	Geographic region, age, pack-years of smoking, current smoking status, energy intake, intake of meat, and intake of fruits and vegetables	Water, milk, soda, coffee, fruit juice
Ros *et al.* 2011 [36]	European	F/M	233,236/513	F: >2046 vs. <1,438 ml/day	8	1.12 (0.86-1.45)	Age at entry, sex and centre and adjusted for smoking status, duration of smoking, lifetime intensity of smoking, energy intake from fat and nonfat sources	Alcoholic beverages, milk and other dairy beverages, coffee, tea, herbal tea, water, fruit and vegetable juices, and soft drinks
						F: 1.15 (0.73-1.81)		
				M: >2,425 vs. <1,735 ml/day				
						M: 1.09 (0.79-1.5)		
Zeegers *et al*. 2001 [37]	Netherlands	F/M	120,852/569	The highest vs. lowest quintile	7	0.91 (0.65-1.29)	Age, sex, number of cigarettes/day, years of cigarette smoking, coffee consumption, and tea consumption	Water, milk, juice, soda and lemonade, alcoholic beverages, coffee, and tea

F, female; M, male.

### Total fluid intake and risk of bladder cancer

**Figure 2:**
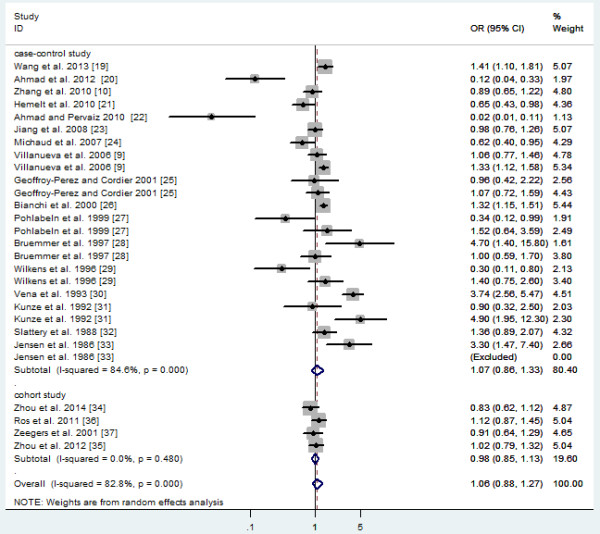
Forest plot of the association between total fluid intake and bladder cancer risk.

Risk estimates for highest versus lowest level of total fluid consumption are shown in Figure 2. The summary OR with 95% CI (1.06 95% CI: 0.88-1.27) of all studies, using a random effects model, showed a statistically significant association between the highest fluid intake and the risk of bladder cancer. There was statistically significant heterogeneity among studies (*P*<0.001, I^2^ = 82.8%).

### Subgroup meta-analysis

To explore the source of the heterogeneity, we conducted subgroup meta-analysis by various study characteristics (Table 3). In an analysis stratified by study design, the summary OR from cohort studies (OR 0.98; 95% CI 0.85-1.13) showed that there was no association for the highest fluid intake with the risk of bladder cancer, but in case-control studies (OR 1.07; 95% CI 0.86-1.33) showed a significant association. When stratified by geographical region, studies conducted in Europe and North America did not change the overall OR of all studies (OR 1.20; 95% CI 1.02-1.42), but there is a statistically significant protective effect of the highest fluid intake on bladder cancer in Asia (OR 0.27; 95% CI 0.10-0.72). When we separated the population-based case-control studies from the hospital-based case-control studies, we found that hospital-based studies markedly changed the summary OR of all studies (OR 0.71, 95% CI 0.44-1.15), in that greater fluid intake was related to decreased bladder cancer risk. Moreover, to avoid inadequate adjustment for tobacco smoking in the included studies, we evaluated the influence of the smoking status. The results showed that the smoking status did not influence the summary ORs substantially (Table 3). Interestingly, we found green and black tea have an inverse association for the highest tea intake with the risk of bladder cancer (OR 0.76, 95% CI 0.66-0.95; OR 0.80, 95% CI 0.65-0.97, respectively), but no statistical significance. The majority of them were not identified as a possible source of heterogeneity among all studies included (Table 3).

**Table 3:** Stratified pooled OR and 95% CIs for the highest vesus lowest level of total fluid intake and bladder cancer risk

**Subgroup**	**Number of studies**	**Pooled OR (95% CI)**	***P* values for interaction or moderation effect between the stratified factor and fluid consumption**
All studies	21	1.06 (0.88-1.27)	<0.001
**Study design**			
Cohort studies	4	0.98 (0.85-1.13)	0.480
Case-control studies	17	1.07 (0.86-1.33)	<0.001
**Gender**			
Female	11	1.00 (0.74-1.35)	0.012
Male	10	1.22 (0.95-1.57)	<0.001
**Geographical Region**			
Europe and America	17	1.20 (1.02-1.42)	<0.001
Asia	4	0.27 (0.10-0.72)	<0.001
Asia*	2	0.78 (0.58-1.06)	0.234
**Publication time**			
After 2000	14	0.92 (0.77-1.10)	<0.001
Before 2000	7	1.51 (0.93-2.47)	<0.001
**Type of control**			
Hospital	8	0.71 (0.44-1.15)	<0.001
Population	13	1.21 (1.01-1.45)	<0.001
**Smoking status**			
Never	6	0.93 (0.73-1.19)	0.471
Ever	6	0.80 (0.62-1.02)	0.032
**Adjustments #**			
≥ 3 factors	14	1.12 (0.94-1.33)	<0.001
< 3 factors	7	0.88 (0.45-1.70)	<0.001
**Type of beverage**			
Water	11	1.05 (0.88-1.25)	<0.001
Coffee	14	1.17 (1.03-1.33)	0.005
Tea	7	0.96 (0.77-1.21)	0.044
Green tea	5	0.76 (0.66-0.95)	0.317
Black tea	4	0.80 (0.65-0.97)	0.291
Alcoholic beverage			
Wine	5	0.90 (0.61, 1.32)	<0.001
Beer	6	1.09 (0.79-1.49)	0.001
Liquor	5	0.98 (0.77-1.24)	0.094
**Number of beverages**			
≥ 5	16	1.19 (0.97-1.46)	<0.001
< 5	4	0.60 (0.34-1.06)	<0.001

*except papers that did not adjust for smoking habits and that calculated total fluid intake based on 3 beverages only.

#adjusted for at least three of the following factors: age, gender, smoking, BMI, education,energy intake and place of residence.

### Qualitative assessment and publication bias

**Figure 3:**
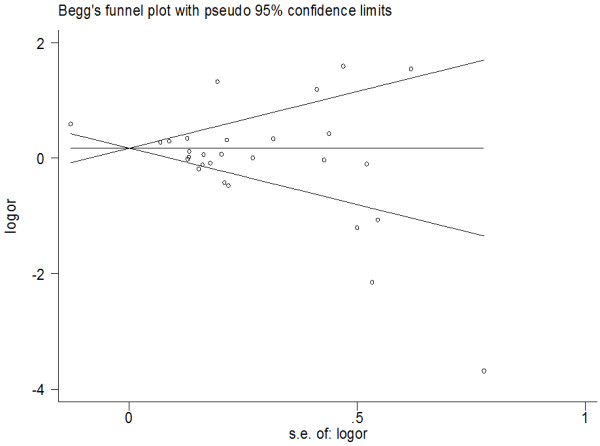
**Funnel plot of studies included evaluating the association between total fluid intake and bladder cancer risk.** (S.E: Standard Error).

The results of qualitative assessment of the studies included in our meta-analysis were shown in Tables 1 and 2. All of them were given a score of 6 to 8 stars. There was no funnel plot asymmetry for the association between total fluid intake and risk of bladder cancer, *P* values for Begg’s adjusted rank correlation test was 0.244, and the Egger’s regression asymmetry test was 0.059, which suggested that there was no evidence of publication bias in this meta-analysis (Figure 3).

## Discussion

Dietary intervention is an attractive, valuable, and noninvasive means to prevent cancer. Fluid intake may impact the development of bladder cancer along with multiple pathways, ranging from carcinogenesis to cellular apoptosis. However, contradictory conclusions were summarized by different research centers. Therefore, we conducted this meta-analysis to assess the potential relationship between total fluid intake and bladder cancer risk, and the summary OR indicated that there was no significant association between them.

To further illustrate the relationship, stratified analyses were performed, and the finding of subgroup analysis of hospital-based case-control studies suggested that higher fluid intake was related to decreased bladder cancer risk. However, meta-analysis of observational studies presented particular challenges because of inherent biases, residual confounding, and chance that might diminish the validity of a systematic review. The potential bias of case-control studies, such as selection and recall bias, might contribute to the discrepancy between case-control and cohort studies. Therefore, considering this condition, caution is needed in interpreting the findings from the meta-analysis.

Several potential mechanisms have been proposed to explain the associations of fluid intake and bladder cancer risk. A higher level of fluid consumption could reduce the contact time between urinary carcinogens and the bladder urothelium through increasing urination frequency, which could reduce the risk of bladder cancer [23,38,39]. We should note that the amount of total fluid ingested and urination frequency may not present a positive correlation among older men because of benign prostatic hyperplasia, which can cause frequent micturition [35]. Furthermore, urination frequency is related to different weather conditions, which may partly explain some of the geographic differences [35].

However, given that fluids may carry carcinogens to the bladder, another hypothesis was proposed to explain the increased risk. An increase in total fluid consumption may extend the bladder wall and introduce the carcinogens to the deeper layer of the bladder urothelium, which inevitably results in the formation of DNA adducts that may induce the critical mutations needed for tumorigenesis [40]. Thus, increased fluid consumption will increase the risk of bladder cancer. A meta-analysis found that high concentrations of arsenic in the drinking water (>50 μg/L) was associated with an increased risk of bladder cancer incidence and mortality [3]. However, the mechanisms of the effect of fluid intake on bladder cancer require further research.

The role of total fluid intake is further complicated because of the possibility that different types of fluid may have different effects on bladder cancer. We evaluated specific fluid items, including water, tea, coffee, and alcoholic beverages. Inconsistent with the previous findings that water intake contributed to a lower risk [10,24], we found that a greater water intake may increase the risk but the relationship is weak. This discrepancy may be due to differences in exposures to chlorine and chlorination by-products [41,42]. Unfortunately, we did not collect information on the source of drinking water because of the limitations of the included studies.

Regarding the role of coffee in bladder cancer, our result suggest a positive correlation between them. Many constituents in coffee could potentially affect the risk of bladder cancer through several biological mechanisms. The main mechanisms, including caffeine, can inhibit the radiation-damaged DNA repair, modify the apoptotic response, and reverse cell-cycle checkpoint function [43,44]. On the other hand, the positive findings could be the result of residual confounding by smoking [36].

It should be fully considered that different regions have different drinking habits, for example, coffee is one of the major beverages consumed in Europe, while tea is one of the most popular beverages in Asia. Our study suggested that high total tea intake decreased the risk of bladder cancer. Previous research found that polyphenols, which are the active ingredient of tea, provided protection against bladder cancer by antioxidant activity [45]. Catechins are the most prevalent polyphenols in green tea. For black tea, the amount of catechin is about one third of that in green tea because of a different process of fermentation. Therefore, we conducted the stratified analysis by type of tea to assess the effect of them, and our results are consistent with previous studies that suggest a significantly inversed correlation between green tea consumption and risk of bladder cancer [46]. Such association was also found in black tea. Nevertheless, the former is more obvious than the latter.

In addition, findings from the study suggested that wine consumption was inversely related to bladder cancer risk because wine contains antioxidant phenolic substances, which provide protective effects against cancer by blocking cell-cycle progression in the G0/G1 phase and inducing apoptosis [47]. However, some authors argued against the association and advocated that wine consumption could be harmful because the primary metabolite of ethanol (acetaldehyde) has been shown to cause damage to the DNA, which makes alcohol a plausible bladder carcinogen [48]. Therefore, further high-quality studies are needed to obtain a better understanding of the effect of wine consumption on bladder cancer.

When researching the effects of fluid intake on the risk of bladder cancer, we should consider the multi-factor interactions. Cigarette smoking is the best established risk factor for bladder cancer. Current smoking increases the risk of developing bladder cancer and quitting smoking markedly decreases the risk. Prevention of cigarette smoking would result in a decrease of 50% of male bladder cancer cases and 23% of female bladder cancer cases [49]. A recent study found that increased time between smoking cessation and diagnosis was related to improved prognosis [50]. Moreover, high fluid intake may be most beneficial to the smokers who had been exposed to a high load of bladder carcinogens, such as urinary caffeine, was inversely related to urinary cotinine [51]. The Nurses’ Health Study found that a 38% reduction in bladder cancer risk was associated with increased total fluid intake among heavy smokers [34]. In our results, we also found greater consumption of fluid reduced the bladder cancer incidence among smokers. Furthermore, we also noted that the associations of fluid intake with bladder cancer risk were negative in all studies published after 2000, whereas this was not true in studies published before 2000. This phenomenon may be related to the improvement of the adjustment for smoking in the past decades. Interestingly, some research found that smoking status may influence the fluid choice [19]. Specifically, smokers tended to have high ingest of total fluid, coffee, and alcoholic beverages. In contrast, smokers were less likely to have a high consumption of tea.

Although there is a causal relationship between cigarette smoking and bladder cancer, the influence of smoking on the risk for superficial or invasive cancer remains unclear. The Nurses’ Health Study found an approximately 53% reduction in invasive bladder cancer risk for the highest total daily fluid intake [34], however, it was not observed to be significantly associated with non-invasive bladder cancer risk. They hold that invasive bladder cancer and non-invasive bladder cancer have distinct molecular profiles, such that fluid intake may only be associated with the more aggressive molecular changes. Nevertheless, results from the EPIC’s (European Prospective Investigation into Cancer and Nutrition) study do not support that total fluid intake is associated with low- and high-risk bladder cancer prognosis [36]. Therefore, further investigation into the impact of total fluid intake on the risk for the different pathological types of bladder cancer is needed.

Several limitations of this meta-analysis should be acknowledged. First, we did not search for unpublished studies or original data, so publication bias maybe inevitable, even though no significant evidence of publication bias was observed. Second, we extracted the risk estimates that reflected the greatest degree of control for potential confounders, but confounding by differences in exposures to disinfection byproducts or other fluid contaminants may introduce an unpredictable bias. Third, the differences in the consumption levels in the lowest and highest categories and the range of consumption, which varied across studies, may be responsible for the heterogeneity among studies in this analysis, and the heterogeneity is inevitable. Meanwhile, our meta-analysis did not consider level of consumption in different countries. Future studies should investigative the different types of fluids and level of consumption in different countries associated with bladder cancer risk.

## Conclusions

In summary, our meta-analysis of 4 cohort and 17 case-control studies suggested that greater consumption of fluid may reduce the bladder cancer incidence in smokers, and may also have a protective effect on bladder cancer in Asian people. However, there was no conclusive evidence on this association because of heterogeneity.

## Abbreviations

CI: Confidence interval; OR: Odds ratio; RR: Relative risk; NSAIDS: Non-steroidal anti-inflammatory drugs; THM: trihalomethane; EPIC: European prospective investigation into cancer and nutrition.

## Competing interests

The authors declare that they have no competing interest.

## Authors’ contributions

YB and HY performed statistical analysis and wrote the manuscript; JL and YT performed literature search and stratified the data; CP and YB provided meaningful discussion key points; PH revised and edited the manuscript. All authors read and approved the final manuscript.
